# Wind as a Driver of Peat CO_2_ Dynamics in a Northern Bog

**DOI:** 10.1007/s10021-024-00904-1

**Published:** 2024-05-23

**Authors:** A. Campeau, H. He, J. Riml, E. Humphreys, M. Dalva, N. Roulet

**Affiliations:** 1 Department of Forest Ecology and Management, Swedish University of Agricultural Sciences, Umeå, Sweden; 2 Department of Geography, McGill University, Montreal, Canada; 3 Department of Sustainable Development, Environmental Science and Engineering, KTH Royal Institute of Technology, Stockholm, Sweden; 4 Department of Geography and Environmental Studies, Carleton University, Ottawa, Canada; 5 Present Address: Department of Geography, University of Montréal, Montreal, Quebec Canada

**Keywords:** Carbon dioxide, peatlands, wind, diffusion, respiration, eddy-covariance, non-diffusive transport, continuous measurements

## Abstract

Excess CO_2_ accumulated in soils is typically transported to the atmosphere through molecular diffusion along a concentration gradient. Because of the slow and constant nature of this process, a steady state between peat CO_2_ production and emissions is often established. However, in peatland ecosystems, high peat porosity could foster additional non-diffusive transport processes, whose dynamics may become important to peat CO_2_ storage, transport and emission. Based on a continuous record of in situ peat pore CO_2_ concentration within the unsaturated zone of a raised bog in southern Canada, we show that changes in wind speed create large diel fluctuations in peat pore CO_2_ store. Peat CO_2_ builds up overnight and is regularly flushed out the following morning. Persistently high wind speed during the day maintains the peat CO_2_ with concentrations close to that of the ambient air. At night, wind speed decreases and CO_2_ production overtakes the transport rate leading to the accumulation of CO_2_ in the peat. Our results indicate that the effective diffusion coefficient fluctuates based on wind speed and generally exceeds the estimated molecular diffusion coefficient. The balance between peat CO_2_ accumulation and transport is most dynamic within the range of 0–2 m s^−1^ wind speeds, which occurs over 75% of the growing season and dominates night-time measurements. Wind therefore drives considerable temporal dynamics in peat CO_2_ transport and storage, particularly over sub-daily timescales, such that peat CO_2_ emissions can only be directly related to biological production over longer timescales.

**Supplementary Information:**

The online version contains supplementary material available at 10.1007/s10021-024-00904-1.

## Highlights

- Wind speed and temperature generate diel cycles in peat CO_2_ concentration.
- Wind can effectively flush CO_2_ out of the peat.
- Wind-driven CO_2_ emissions have implications across multiple aspects of ecosystem studies.

## Introduction

Northern peatlands exchange CO_2_ continuously with the atmosphere, which results in a small but persistent global C-sink (Treat and others 2019). Conceptually, this exchange takes place between two compartments: the atmosphere and the biosphere (Baldocchi and Monson 2014; Loisel and others 2020). The atmosphere (that is, boundary layer) is mostly turbulent and acts as a recipient of biogenic CO_2_. The biosphere, incorporating the canopy and the soil, acts as both a sink and a source of CO_2_, through the counteracting action of plant photosynthesis and respiration from both soil and plants. Momentum transfer typically breaks down somewhere below the canopy due to the surface friction drag, making the biosphere mostly free of turbulence. The lack of turbulence below this boundary leaves only the steady and slow process of molecular diffusion along a concentration gradient as the driver of vertical CO_2_ transport through soils and into the atmosphere (Lerman 1979; Pumpanen and others 2003). Based on this compartmentalization of the atmosphere and biosphere is a suit of ecological concepts (Orchard and Cook 1983; Davidson and Janssens 2006; Barba and others 2018), process-based models (Parton and others 2010; Smith and others 2014; He and others 2021) and measurement techniques (for example, soil chambers, eddy covariance) (Baldocchi 2003).

Peat CO_2_ emissions should be at a steady state with CO_2_ production when vertical gas transport is driven by molecular diffusion (Pumpanen and others 2003; Rey 2015; Barba and others 2018). Together, peat CO_2_ production and emission should therefore respond mostly to ambient temperature changes, which determine production (Lloyd and Taylor 1994; Wu and others 2012; Yvon-Durocher and others 2012), and plant C uptake, which alters the CO_2_ gradient between the peat and the atmosphere and therefore the rate of diffusion. However, intermittent non-diffusive transport processes could override molecular diffusion and create phase shifts in peat CO_2_ emissions, with periods of storage and delayed emission relative to production. These non-diffusive transport processes can operate across multiple timescales and include convective fluxes, such as thermal convection (Spohn and Holzheu, 2021), pressure pumping (Massman 2006) and buoyancy flow (Rappoldt and others 2003), turbulent diffusion (Campeau and others 2021) and advective fluxes, such as soil venting (Hirsch and others 2004; Redeker and others 2015; Moya and others 2022). These processes may not considerably alter the estimate of the annual C balance of ecosystems, but nonetheless represent important underlying mechanisms that determine the magnitude and timing of soil CO_2_ emission at a shorter timescale. Terrestrial ecosystems with the highest potential for non-diffusive transport processes are soils with high porosity, strong exposure to wind and where large temperature fluctuations occur. While all of these criteria apply to northern peatlands, physical gas transport processes in peat have received far less attention than biological drivers of peat CO_2_ production and emissions (Pumpanen and others 2003; Phillips and others 2011; Rey 2015).

Here, we explore the temporal dynamics in peat pore CO_2_ based on in situ continuous half-hourly measurements of CO_2_ concentration at different depths in the unsaturated peat of raised bog (Mer Bleue bog, Canada). Temporal changes in peat pore CO_2_ concentration reflect the continuously developing balance between local CO_2_ production, transport and emission. We hypothesize that because of the influence of non-diffusive transport processes, peat pore CO_2_ concentrations will exhibit little temperature sensitivity, particularly over short timescales. To help identify the physical controls over peat pore CO_2_ store over time, we complemented our analysis with above-ground CO_2_ concentration measurements (0.1–2.6 m above ground), eddy covariance (EC)-derived CO_2_ fluxes, together with peat temperature, water table depth and meteorological measurements, such as precipitation, atmospheric pressure and wind speed.

## Methods

### Site Description

Mer Bleue is a 28 km^2^ domed ombrogenic, oligotrophic bog located near Ottawa, Canada (45.41°N, 75.48°W). The area underwent bog formation starting approximately 7000 years ago (Roulet and others 2007). The depth of the accumulated peat ranges between 0.3 m at the margins and 5–6 m on average near the centre of the bog. The local climate is temperate and humid, with a mean annual air temperature of 6.0 °C. The annual precipitation at the site is 943.5 mm, with ~ 25% falling as snow. The research site area has a hummock–hollow microtopography with a mean elevation difference of 0.25 m between hummock tops and hollow bottoms (Lafleur and others 2003). Hummocks cover approximately 70% of the bog, and the remaining 30% is hollows. The bog has a relatively thick unsaturated zone. The seasonal water table fluctuates between 20 cm and as much as 80 cm below the hummock surface during the driest summers and averages 40 cm below the hummock surface over the growing season (Teklemariam and others 2010; He and others 2023). The peat porosity in the hummocks ranges from 99% near the surface to 94% at 40 cm depth (Dimitrov and others 2010). Moss vegetation at the study location is almost completely dominated by *Sphagnum capillifolium* and *Sphagnum magellanicum*. The vascular plant community of the overstory is dominated by ericaceous shrubs (*Chamaedaphne calyculata*, *Kalmia angustifolia*, *Rhododendron groenlandicum* and *Vaccinium myrtilloides*) along with some sedges (*Eriophorum vaginatum*) and forb (*Maianthemum trifolium*) (Bubier and others 2006). The peatland also supports a few scattered tree species such as (*Larix laricina, Betula populifolia* and *Picea mariana*).

### Peat Pore CO_2_ Concentration, Temperature and Water Level Measurements

Half-hourly measurements of CO_2_ concentration (ppm) in peat were taken using the Vaisala CARBOCAP GMP220 and GMP221 non-dispersive infrared (NDIR) CO_2_ sensors. The CO_2_ sensors were deployed horizontally at depths of 5, 10, 20 and 40 cm below the ground surface in the hummock and 5 and 10 cm below the ground surface in the hollow. Each sensor was enclosed inside an expanded polytetrafluoroethylene (PTFE) sleeve to ensure that the sensor was protected from water but remained permeable to gases. Sensors were deployed in 2008, but measurements were collected from June to December 2009 to ensure recovery of the site after instrumentation. The sensor measurements represent a gas phase concentration of CO_2_ that is in equilibrium with any peat pore air or water, which is variable in time according to soil moisture conditions. Soil temperature was measured using thermocouples at depths of 1, 5, 10, 20 and 40 cm in one hummock and one hollow using arrays of copper–constantan thermocouples embedded in wood dowels. Both CO_2_ and temperature measurements were recorded at 5 min intervals, averaged every 30 min and stored on a data logger (CR21X, Campbell Scientific, UT, USA).

Water table position was measured in two wells (one in a hollow and one in a hummock) using a float and counterweight system attached to a potentiometer. Frequent manual observations were used to verify the water level measurements and were expressed as the average water level depth below the hummock surface. CO_2_ concentrations are typically higher and more stable in porewater than in air-filled pores, with colder and more constant temperatures (Blodau and others 2007; Campeau and others 2021). Although water table records indicate that the depth of the unsaturated zone varied from 25 to 45 cm below ground throughout the study periods (Supplementary Figure S1), the CO_2_ concentration and temperature measurements at − 40 cm indicate that the sensor was never fully submerged below the water table. It is worth noting that the surface of this peatland is highly variable due to the hummock and hollow microforms, such that ground surface reference for the water table and our CO_2_ concentration profile may differ slightly. The average water table position of 34 cm below the ground surface during the study year was similar to the long-term average (Supplementary Figure S1, He and others 2023).

### CO_2_ Exchange and Environmental Variables

This peatland has been continuously monitored for CO_2_, energy (latent and sensible heat) and momentum fluxes using an EC system since 1998 (Lafleur and others 2003). During the study period, the EC system consists of a three-dimensional sonic anemometer (model R3-50 Solent, Gill Instruments, Lymington, England), a closed-path infrared gas analyser (IRGA, model 7000, LI-COR Inc., Lincoln, NE, USA) and fine wire thermocouple (25 mm diameter). The sonic anemometer and intake for the IRGA were mounted 2.6 m above the bog surface on the 8 m tower. The net CO_2_ exchange (NEE) between the atmosphere and the bog surface is computed as the sum of the 30-min covariance of the CO_2_ mixing ratio and vertical velocity and the rate of change in CO_2_ concentration measured at the height of the EC instruments. Night-time fluxes are removed from the record when friction velocity is less than 0.1 m s^−1^. NEE was partitioned into component fluxes of ecosystem respiration (ER) and gross primary production (GPP) using temperature and light response relationships. A detailed description of all flux data handling and quality control procedures is provided by Roulet and others (2007).

A smaller tower close to the soil CO_2_ sensors was equipped with a series of intake tubes at 0.1, 0.2, 1.2 and 2.6 m leading to an LI-6262 closed-path IRGA (LI-COR). A pump and solenoid valve system drew air into the IRGA from each intake for 2 min. CO_2_ readings from the first minute were discarded to account for line flushing. The readings from the second minute were recorded on a 21X data logger (CSI) to determine the 30 min average CO_2_ concentration (ppm) from each of the four levels.

Auxiliary environmental measurements were taken in support of the CO_2_ concentration profile and EC measurements including radiation (long and shortwave radiation, model CNR1 Kipp & Zonen, Delft, the Netherlands), air temperature and relative humidity (model HMP35 probe, Campbell Scientific, Logan, UT, USA) and wind speed, which is primarily obtained from the sonic anemometer, but occasionally gap-filled with cup wind speed (model 20,120, R.M. Young Company, MI, USA) measured 2.0 m from the bog surface after correction for height differences.

### CO_2_ Storage Calculation

The total amount of CO_2_ stored in the peat pores and above ground was estimated at 30 min intervals over a 1 m^2^ area. The volumetric pore space at each peat depth was estimated based on bulk density measurements described by Dimitrov and others (2010). The peat surface was assumed to correspond to the top of the moss capitulum, while the lower depth of the unsaturated zone fixed at 40 cm, which corresponds to the permanently unsaturated zone during our study period. The amount of CO_2_ stored in the air was calculated from the height of the EC system (2.6 m) to the peat surface. Concentrations of CO_2_ (ppm) were first converted to density (g C cm^−3^) using the ideal gas law according to continuous atmospheric pressure and temperature measurements at each depth below ground and into the air. Densities were linearly interpolated between the concentration measurement locations. The total CO_2_–C stored in the peat pores and air above ground was obtained by the sum of the volume-weighted CO_2_–C density at each layer. Wind could move gas phase CO_2_ faster than CO_2_ dissolved in soil moisture, but we consider that equilibrium between those two phases occurs rapidly in the peat. Therefore, the estimate of peat pore CO_2_ store considers the full storage without distinction between the gas and dissolved phases.

### Effective Diffusivity Calculations

The effective diffusion coefficient (*D*_eff_, cm^2^ s^−1^) was derived based on Fick’s first law of diffusion, as follows:

1 $$ D_{{{\text{eff}}}} = {\text{ FCO}}_{{{2} }} / \, \left( {\Delta {\text{CO}}_{{2}} /\Delta {\text{z}}} \right). $$

where FCO_2_ represents the CO_2_ emission from the peat to the atmosphere. This FCO_2_ is a result of saprotrophic and mycorrhizal (that is, heterotrophic (HR) and autotrophic respiration (AR)) from the belowground plant parts. Since there was no direct measure of FCO_2_ at the Mer Bleue site, we roughly estimated FCO_2_ as representing on average 48.5%, with a minimum of 20% and a maximum of 80% of the total EC-derived ecosystem respiration (ER) measurements, and expressed this 30 min flux as g cm^−2^ s^−1^. This estimate of FCO_2_ is based on the partitioning of HR and AR at this site, which was made using a combination of dark and light soil chamber measurements over plots with variable degrees of vegetation cover, ranging from intact to completely clipped (Rankin and others 2022). The ∆CO_2_ is the difference between CO_2_ density (g cm^3^) in the air (10 cm above ground) and the peat pores (10 cm below ground). The ∆z is the distance between those two depths, which is 20 cm. The *D*_eff_ is therefore the rate of diffusion needed to sustain the FCO_2_ while maintaining the CO_2_ gradient between the air and peat compartments.

Non-diffusive transport processes are considered to occur if *D*_eff_ exceeds the coefficient of molecular diffusion in the peat pore space. Because molecular diffusion is not directly measured, we roughly estimate it as ${{\text{D}}}_{{\text{o}},\text{air}}$ (cm^2^ s^−1^), the temperature-dependent molecular diffusion coefficient of CO_2_ in the air according to Lerman (1979).

2 $${D}_{{\text{o}},\text{ air}}={\left(0.1325+0.00009\times {T}_{s}\right)}$$

where *T*_*s*_ is the temperature of the soil at a specific depth. Note that this will be a slight overestimate as peat porosity is not 100%, but instead ranges from 99% near the surface to 94% at 40 cm below the ground surface (Dimitrov and others 2010).

### Temperature Dependence of Peat Pore CO_2_ Store

A least square linear regression model was applied to the peat CO_2_ store as a function of the average peat temperature (*Ts*) while selecting only observations with the lowest measurable wind speed (< = 0.3 m s^−1^) measured at 2.6 m above ground (range 0 to 7.8 m s^−1^ throughout the full measurement period) (Supplementary Figure S2). This model estimates the plausible magnitude of peat pore CO_2_ store change over time as a function of soil temperature only in the absence of wind transport:

3 $$ {\text{CO}}_{{2}} {\text{Store }}\left( {{\text{g m}}^{{ - {2}}} } \right) \, = \, 0.0{9 }\left( { \pm 0.00{2}} \right) \times 0.0{1 }\left( { \pm 0.000{12}} \right)T_{S} $$

*p*-value < 0.0001, *R*^2^ = 0.56, *n* = 3070.

### Spectral Decomposition and Statistical Analysis

Spectral decomposition examines a signal in the frequency domain and utilizes the Fourier transformation of the original time domain representation. The approach separates the inherent fluctuations of a signal into cyclic patterns and provides information on the importance of specific frequencies in the time series. Here, the magnitudes of the fluctuations were evaluated as a function of frequency using the power spectral density, which can be related to the variance of the time series (Stoica 1997; Wörman and others 2017). Thus, the relative importance of specific intervals of periodicities was obtained by normalization of the cumulative distribution function of variances. More details on the spectral decomposition approach using a similar dataset can be found in Riml and others (2019). Wavelength coherence plots were done to assess and visualize the coherence between peat pore CO_2_ store and wind speed or peat temperature across multiple timescales. These calculations were performed using the *R* package (biwavelength) and repeated 1000 times.

Kendall ranked correlation was performed to determine the strength of the correlation between different time series data. Locally weighted least squares (Loess) regression was used to identify the relationship between peat pore CO_2_ store and wind speed. Least square linear regression models were performed on the ∆CO_2_ peat–air (ppm) and wind speed, and *D*_eff_ and wind speed. Figures were generated using packages from the tidyverse (Wickham and others 2019). Analyses were performed using the *R* Core Team (2022) (R: A language and environment for statistical computing. *R* Foundation for Statistical Computing, Vienna, Austria. URL http://www.R-project.org/).

## Results

**Figure 1:**
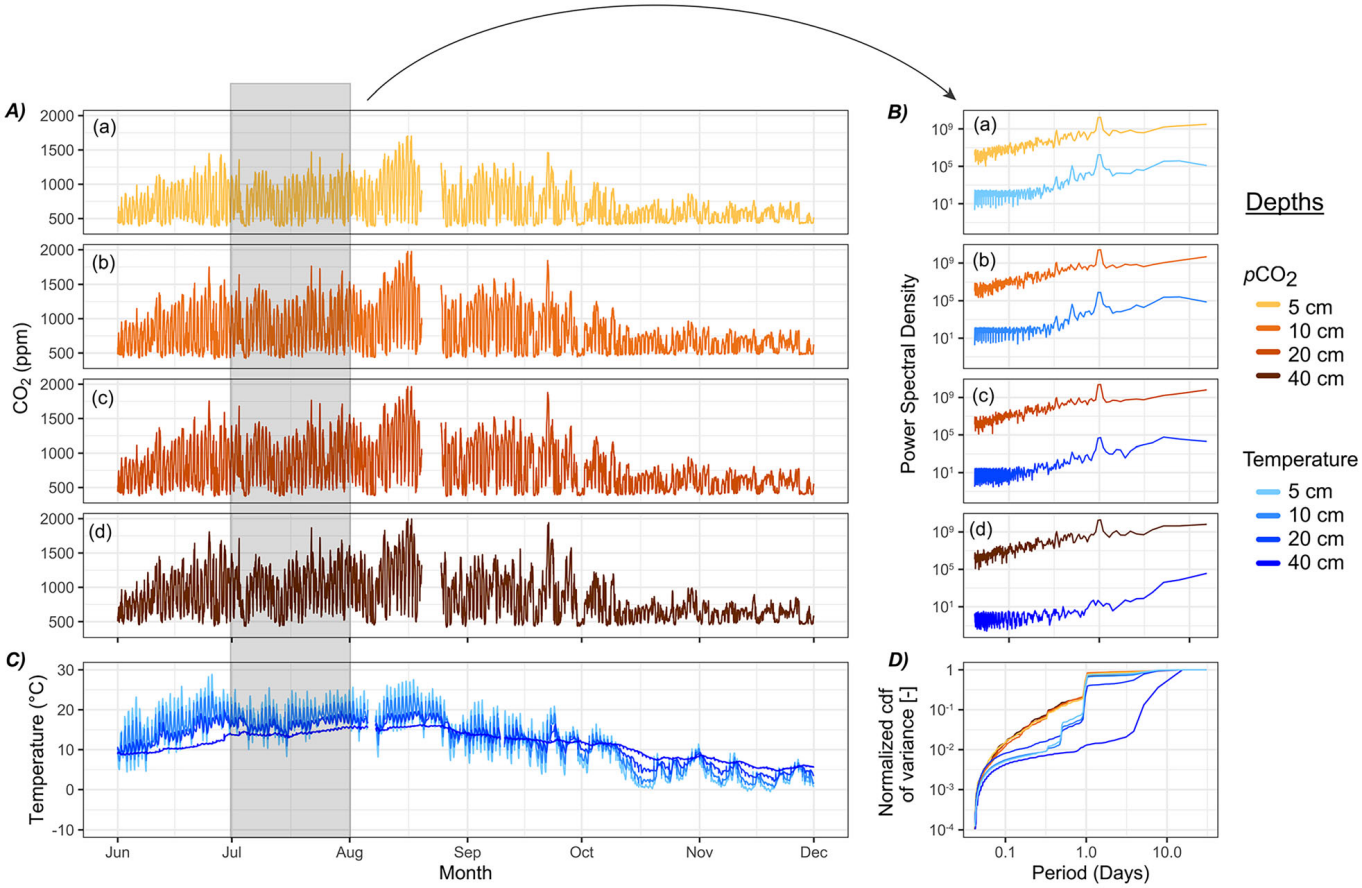
Half-hourly measurements of peat pore **A** CO_2_ concentration with depths divided into four sub-panels (**a** 5 cm, **b** 10 cm, **c** 20 cm and **d** 40 cm) and coloured into orange shades, with increasing depth corresponding to darker shades) and **C** temperatures (same depths as for CO_2_ concentration, but superimposed and in blue shades) along the vertical peat profile between June and December 2009. Panels **B** and **D** present the same data, but in the frequency domain and for July exclusively, with **B** showing the power spectral density for CO_2_ concentration and temperature at each depth with the same colour coding and sub-panelling as in **A** and **D**, and in their cumulative distribution function of variance against the period length in days for all CO_2_ and temperature measurements superimposed.

Peat pore CO_2_ concentration varied from 372 to 1996 ppm across all four depths (5, 10, 20 and 40 cm from the ground surface) between June and December 2009 (*n* = 8544 per depth) (Figure 1A). The CO_2_ was well mixed across the different peat layers, as indicated by the close positive correlation between each depth (*r* ranging from 0.99 to 0.92, Supplementary Figure S3). Spectral decomposition analysis indicated that a persistent 24 h signal dominates the peat pore CO_2_ concentration times series at each depth (Figure 1B). In fact, between 68 and 83% of the cumulative variance in the CO_2_ concentration time series at each depth took place in the periodicities below 24 h over July (Figure 1D). The amplitude of the diel fluctuations in peat pore CO_2_ was larger during the summer months (for example, average in July was 840 ppm, equivalent to 0.18 g C m^−2^) than in the autumn (for example, average in October was 382 ppm, equivalent to 0.08 g C m^−2^).

**Figure 2:**
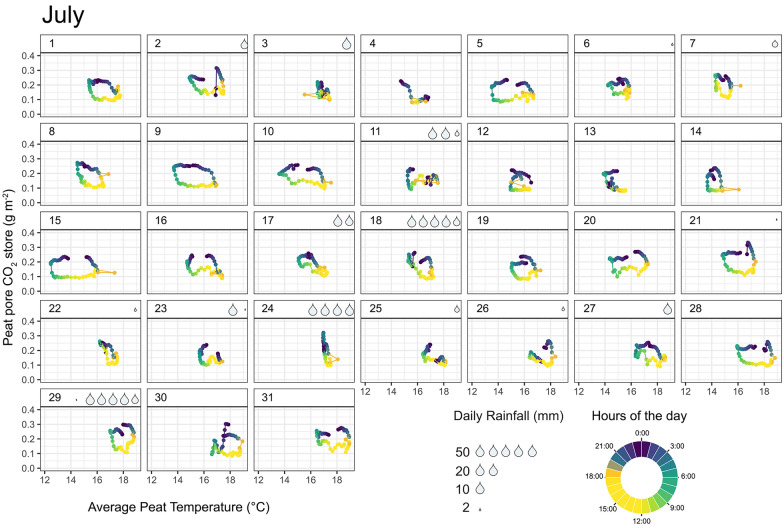
Scatterplot of the total peat pore CO_2_ store (g m^−2^) against the average peat temperature in the top 40 cm peat column. Each panel represents a different day in July 2009 (*n* = 31 panels). Each point represents a half-hourly measurement and is coloured by the time of the day (morning = green, daytime = yellow, dawn = orange, night-time = indigo) and linked together by a line of the same colour gradient (*n* = 48 points per panel). Water drops in the top right corner of each panel illustrate the total precipitation received that day.

For illustration, we narrowed down parts of our analysis to July because this corresponds to a period of the year with the highest biological CO_2_ production and where our data were most complete. During that month, daily peat pore CO_2_ concentration at all four depths attained their minimum in the middle of the day (around 13:00) while the maximum CO_2_ generally occurred in the middle of the night (around 01:00) (Figure 1B). The temperature for the near-surface peat (5 and 10 cm) also followed a diel cycle, but the peat temperatures were mostly constant in the deeper peat (20–40 cm) (Figure 1B). Near-surface peat temperatures reached their lowest value in the morning (around 5–7:00) and their highest value in the late afternoon (between 15 and 17:00). The diel cycle in peat temperature was therefore leading that of CO_2_, by about 10 h at –5 cm and 8 h –10 cm, respectively (Supplementary Figure S4). There was also a regular 12 h signal in the near-surface peat temperature, which was absent in the CO_2_ time series at the same depth and disappeared in the deeper peat horizons (Figure 1). In deeper peat, CO_2_ fluctuated widely and regularly at the daily scale, despite near-constant local temperatures (Figure 1, Supplementary Figure S4-5). As a result, there was a considerable time lag between the diel cycles in peat pore CO_2_ and the average peat temperature (Figure 2, Supplementary Figure S4).

The peat pore CO_2_ store, at 30 min intervals and in the top 40 cm of the peat, averaged 0.15 g C m^−2^ and varied from 0.1 to 0.36 g C m^−2^ during June to December. The relationship between peat pore CO_2_ store and peat temperature revealed a recurrent diel anticlockwise hysteresis loop instead of a linear or power relationship (Figure 2). The peat pore CO_2_ store generally decreased throughout the morning, along with falling peat temperature, and increased throughout the evening with rising peat temperature (Figure 2). However, these periods of rapid change were separated by periods of prolonged stability, mostly throughout the day and night, where the peat pore CO_2_ store remained, low and high, respectively, despite changing temperatures (Figure 2). As a result, the peat pore CO_2_ store was about 3 times larger during the night than during the day for a similar range in temperature. On days following high rain events, the hysteresis loops in peat pore CO_2_ store often collapsed with both temperature and CO_2_ store being more stable throughout the day (Figure 2).

**Figure 3:**
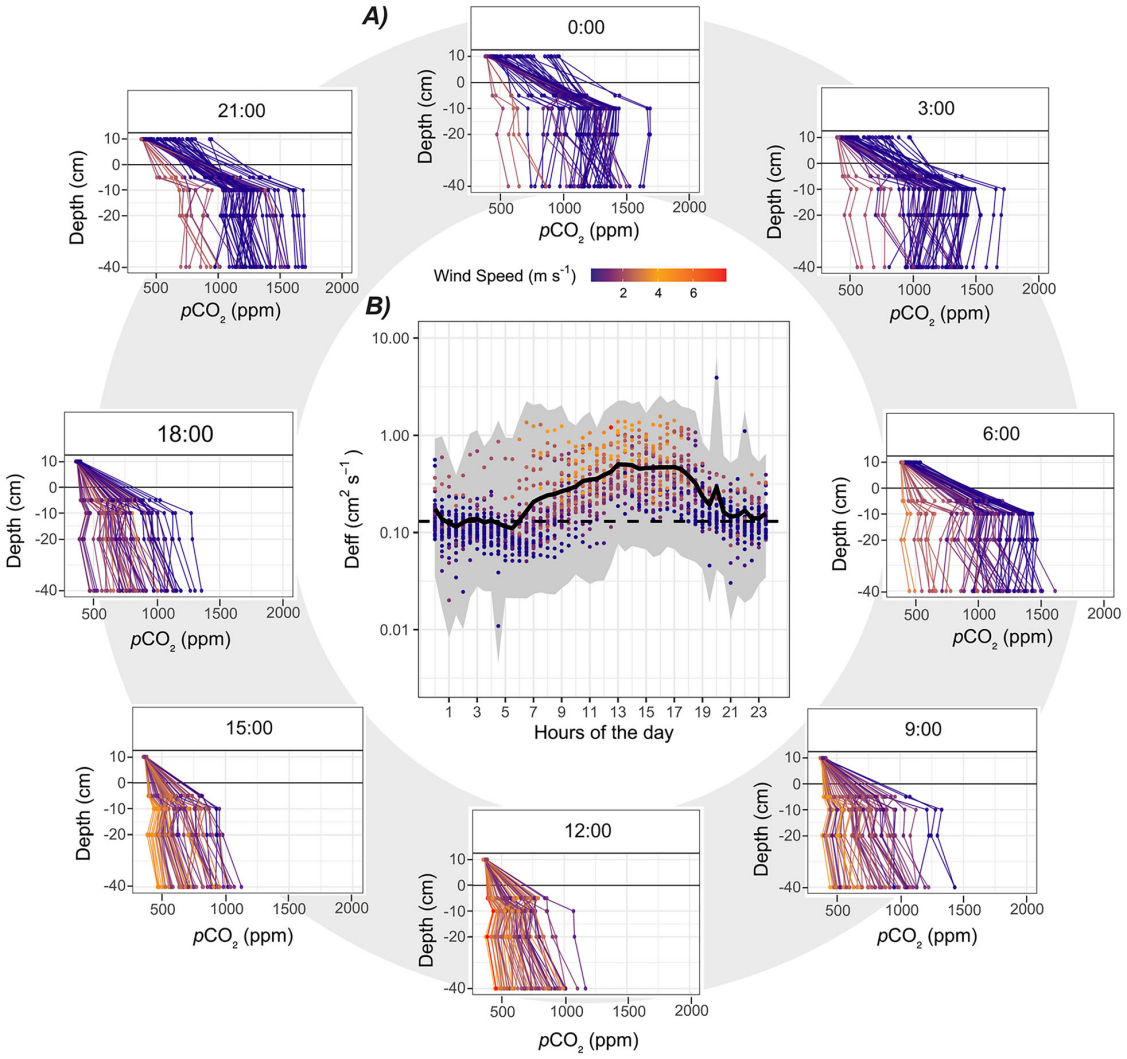
In **A** depth gradient of CO_2_ (ppm) from 10 cm above the peat surface to 40 cm below. Each sub-panel represents a different hour of the day, at 3-h intervals (8 panels). Connected dots in each panel represent a different day in July and are coloured by wind speed, with warmer colours indicating stronger winds. In **B**, the effective diffusion coefficient (*D*_eff;_), on a log scale, against the hours of the day with each dot represents an individual half-hourly measurement coloured by wind speed; the thick black line marks the average *D*_eff_ (assuming FCO_2_ represents 48% of ER), while the grey ribbon marks the range in *D*_eff_ (assuming FCO_2_ represents 20 or 80% of ER). The horizontal dashed line in **B** marks the diffusion coefficient for molecular diffusion (Equation 2). Points falling above this threshold indicate additional non-diffusive transport processes.

**Figure 4:**
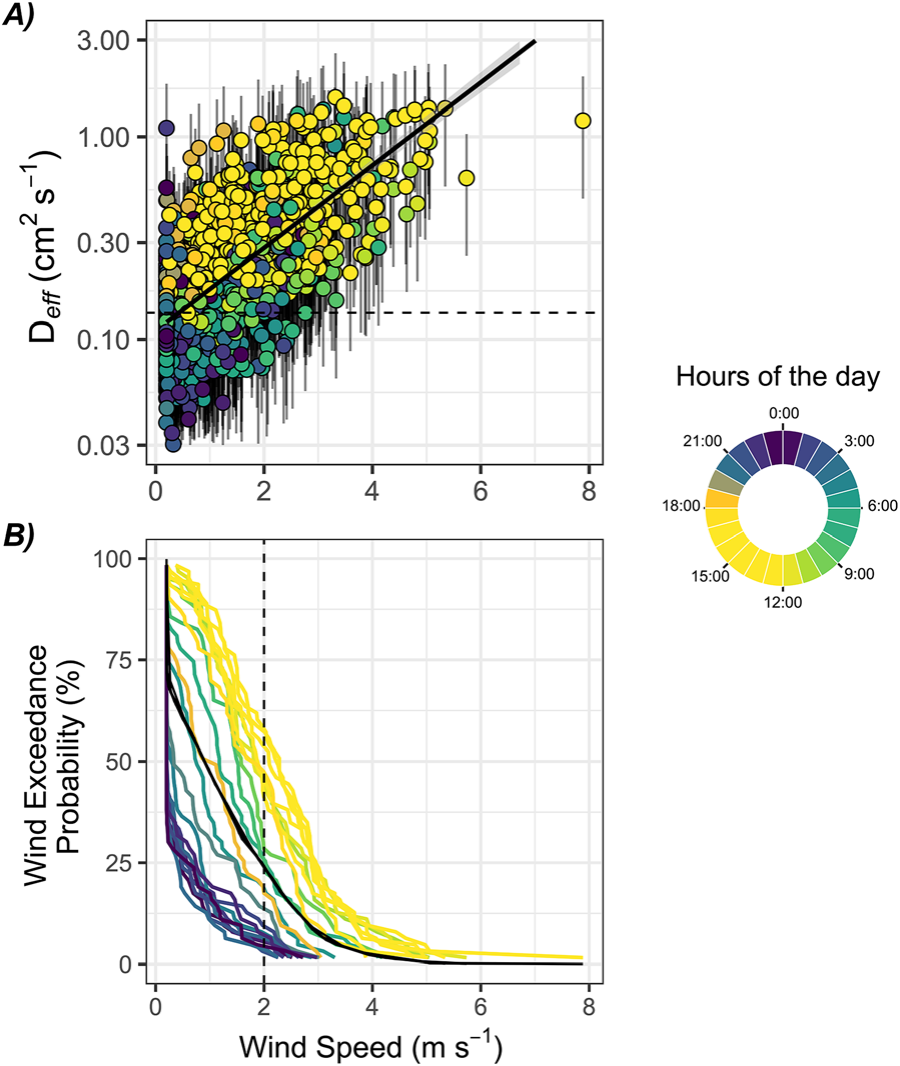
Scatterplots of the effective diffusion coefficient (*D*_eff;_ cm^2^ s^−1^), on a logarithmic scale, against the wind speed (m s^−1^), with each circle coloured by the hours of the day in July 2009 and lines representing the range in *D*_eff_ based on variable FCO_2_ contributions (that is, 20–80% to ER fluxes). In **A**, the horizontal dashed line marks the molecular diffusion coefficient based on Equation 2. In **B**, wind exceedance probability distribution for each hour of the day (coloured lines) and averaged daily (black line). The dashed line marks the 2 m s^−1^ arbitrary threshold identified in (Redeker and others 2015) and where most of the apparent half-hourly change in peat CO_2_ store and *D*_eff_ exceeding molecular diffusion occurs in our data.

There was a steep and persistent positive gradient in CO_2_ concentration between the near-surface peat pores (–10 cm) and the air above ground (+ 10 cm) (∆CO_2_ peat–air), which confirmed that the peat is a continuous source of CO_2_ to the atmosphere. This gradient averaged + 462 ppm across July and also exhibited a recurrent diel cycle. The ∆CO_2_ peat–air was lower during the daytime (average + 302 ppm between 7:00 and 17:00) and increased during night-time (average + 636 ppm, between 21:00 and 5:00), which corresponded with periods of higher and lower wind speed, respectively (Figure 3A). The effective diffusion coefficient (*D*_eff_) varied from 0.012 to 3.2 cm^2^ s^−1^ (Equation 1) and also followed a diel cycle with values near molecular diffusion at night (0.132–0.135 cm^2^ s^1^ (Equation 2)), but increasing almost one order of magnitude during the day (Figure 3B). There was a significant positive relationship between the half-hourly *D*_eff_ and wind speed throughout July (D_eff_ = 0.09 (± 0.008) + WS × 0.15 (± 0.0046), *p* < 0.0001, *R*^2^ = 0.40) (Figure 4).

Low *D*_eff_ and wind speeds, often prevailing at night, allow the CO_2_ to build up in peat pores, despite the cooling of the peat and likely fading local CO_2_ production (Figures 2 and 3). Wind rising the following morning flushes CO_2_ out of the peat pores faster than local production can supply, thus leading to a rapid decrease in peat pore CO_2_ store (Figures 2 and 3). This low CO_2_ store is then maintained throughout the day because high winds instantaneously flush out CO_2_ produced in the unsaturated peat (high *D*_eff_) (Figures 2 and 3). Once *D*_eff_ and wind speed decrease in the evening, CO_2_ builds up rapidly once again, enhanced by the heat accumulated in the peat throughout the day, likely boosting local CO_2_ production (Figures 2 and 3). This cycle is then repeated on each subsequent day to a variable degree. The days with stronger winds (higher *D*_eff_) had lower ∆CO_2_ peat–air and peat CO_2_ store compared to days with lower wind speeds for the same time of the day (Figure 3).

**Figure 5:**
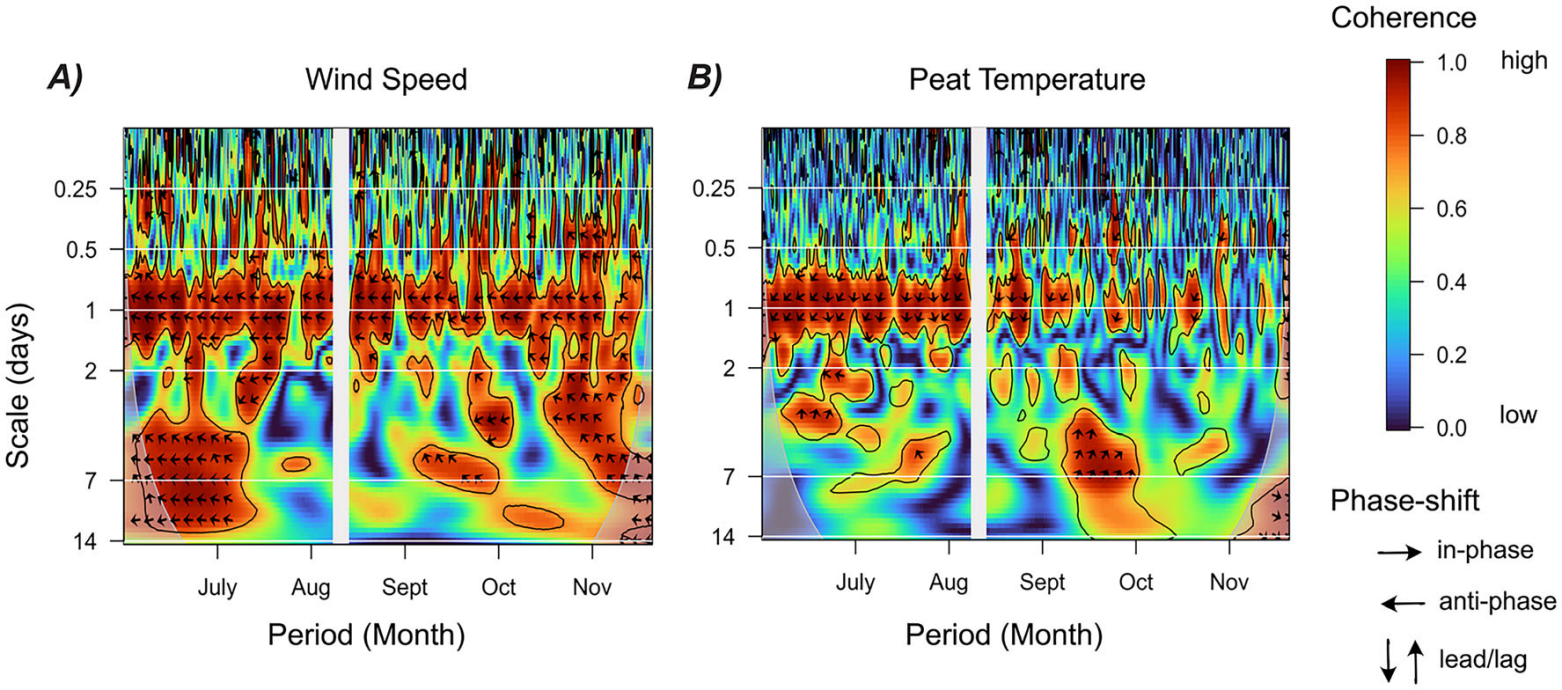
Wavelet coherence plot between half-hourly peat pore CO_2_ store and **A** wind speed at 2.6 m above the ground surface and **B** average peat temperature from 5 to 40 cm below the ground surface. The figures represent a matrix with colours indicating the strength of the coherence between the two time series across different timescales (y-axis; 6 h to 2 weeks) over the full measurement period (x-axis; June–Dec. 2009). Warm colours, like red, indicate a high coherence (for example, high synchronicity), while low coherence is represented by cold colours like blue (for example, low synchronicity). The arrow's direction indicates the phase shift in the coherence between the two time series: right = in phase (that is, the two time series fluctuate together with both peaking at the same time), left = anti-phase (that is, the two time series fluctuate in opposite ways with one peaking while the other bottoms down). The grey area indicates periods of missing data.

Wavelength coherence analysis indicated a higher and more consistent coherence between the peat pore CO_2_ store with wind speed than with peat temperature (Figure 5). The coherence between peat CO_2_ store and wind speed was strongest around the daily timescale (1 day) and consistent throughout the full study period (June to December) (Figure 5A). There were also periods of high coherence at longer timescales (for example, > 1 day), especially in July, October and December. There was an anti-phase lag (left pointing black arrows) between the peat pore CO_2_ store and wind speed, indicating that wind speed peaked when the peat pore CO_2_ store bottomed down (Figure 5A). This phase shift was consistent across all timescales (16 h to 10 days). In comparison, the coherence between peat pore CO_2_ store and peat temperature was also strong around the daily timescale but faded away in the autumn (October–December) (Figure 5B). Contrary to wind speed, the phase shift between peat pore CO_2_ store and temperature indicated that daily peaks in peat temperatures led the daily peaks in peat pore CO_2_ store (downward-pointing black arrows) and this phase shift was not consistent across timescales (Figure 5B).

**Figure 6:**
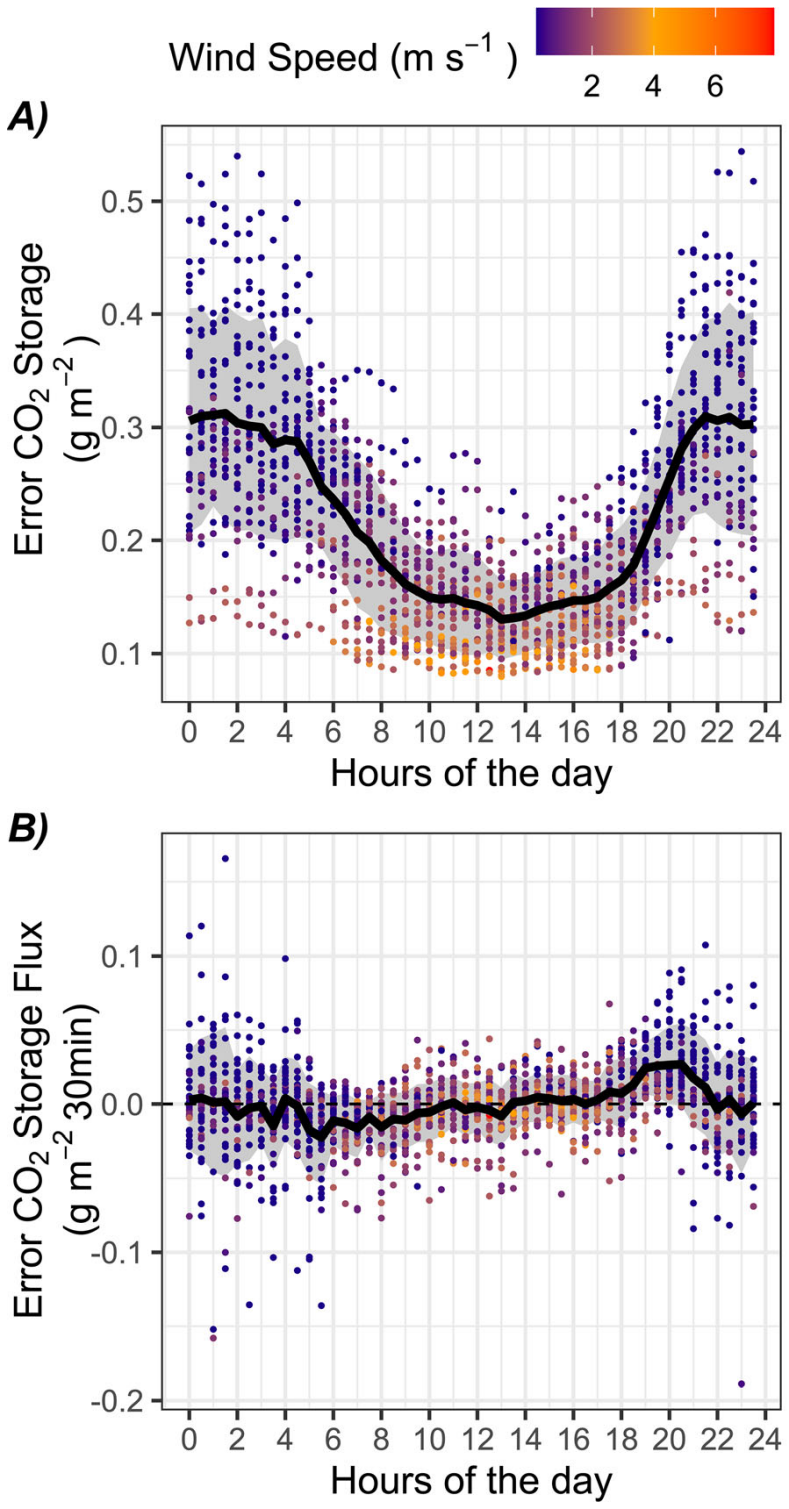
**A** Distribution of the error in half-hourly CO_2_ storage in air or air + peat and **B** the corresponding error in CO_2_ storage flux (g m^−2^ 30 min^−1^) between both estimates (air vs air + peat). Dots are coloured by the wind speed and the dotted line in **B** marks 0, which corresponds to no error between the two estimates. The thick black line represents the average error over July, while the grey area represents the standard deviation.

The full amplitude of the daily changes in CO_2_ store in the top 40 cm of the peat column ranged from 0.003 to 0.31 g C m^−2^, with an average of 0.14 g C m^−2^ across the full study period. In comparison, the amplitude of the modelled daily changes in peat pore CO_2_ store based on temperature sensitivity alone was 0.002–0.075 g C m^−2^, with an average of 0.031 g C m^−2^ over the full study period (Equation 3, Supplementary Figure S2). The bias in CO_2_ storage when accounting only for the atmosphere, or both the atmosphere and surface peat, was on average 0.3 g m^−2^ at night and 0.1 g m^−2^ during the day (Figure 6A). However, the changes in CO_2_ storage over time (that is, storage flux) between those two estimates were mostly random throughout the day (Figure 6B).

## Discussion

### Influence of Wind Speed and Temperature on Peat Pore CO_2_ Store

Our data indicate that wind speed, soil moisture and temperature work in synergy to create large fluctuations in peat CO_2_ store across multiple timescales; from the sub-daily (Figures 1, 2 and 3) to daily and seasonal timescales (Figure 5). At the sub-daily timescale, wind speed rather than temperature drives temporal variations in peat CO_2_ concentration, with diel changes in wind speed prohibiting the accumulation of CO_2_ in the peat beyond a few consecutive hours at night-time. The temperature sensitivity of peat pore CO_2_ store at the sub-daily timescale gave rise to a recurrent anticlockwise hysteresis loop (Figure 2), which typically indicates that secondary processes, including non-diffusive gas transport processes, interplay with local biological CO_2_ production (Phillips and others 2011; Zhang and others 2015; Koschorreck and others 2022). The shape of this hysteresis loop, however, changed considerably following rain event (Figure 2), indicating that rising soil moisture could dampen these non-diffusive transport processes at the sub-daily and daily timescale. We estimate that the observed diel fluctuations in peat CO_2_ store are on average 5 times larger than would otherwise be under strict temperature sensitivity (Equation 3, Supplementary Figure S5). The effect of wind also extends beyond the sub-daily timescale to longer periods, with windier days storing less CO_2_ in the peat compared with calmer days (Figures 3, 4 and 5).

The effect of wind on peat CO_2_ transport is possibly greatest in ecosystems where soil volumetric pore space is large, which is characteristic of most peatlands. Even though CO_2_ transport in water is orders of magnitude slower than in air (Lerman 1979), kinetic energy from the atmosphere has been shown to penetrate deep into the peat porewater of a fen, where it can lead to large seasonal variations in peat porewater CO_2_ store (Campeau and others 2021). The calculated effective diffusion coefficient (*D*_eff_) indicates that molecular diffusion would be too slow and steady to explain the peat CO_2_ emissions (FCO_2_) estimated at this site (that is, *D*_eff_ exceeds 0.135 cm^2^ s^−1^, Figures 3B and 4A). Instead, the *D*_eff_ varies according to wind speed and ranges from a level close to molecular diffusion (mostly at night-time) to an order of magnitude above that level (mostly during daytime) (Figure 3B). Soil venting has been observed in peatlands using experimental wind tunnels on the field (Redeker and others 2015) or controlled laboratory experiments (Poulsen and others 2017; Bahlmann and others 2020). To our knowledge, this study is the first to reveal the synergic effect of wind speed, temperature and soil moisture on peat CO_2_ concentration based on in situ continuous measurements in a pristine peatland. A similar effect of wind on soil CO_2_ transport has been identified in forest litter using a similar methodology to ours (Hirsch and others 2004). Soil venting was also identified in EC measurements of CO_2_ flux from desert ecosystems where vegetation is sparse (Moya and others 2022). Previous studies at this bog also noted the influence of wind on soil chamber measurements (Lai and others 2012) and NEE measurements during the non-growing season (Rafat and others 2021). The synergic influence of wind speed and temperature on peat pore CO_2_ store is most dynamic in the range of 0–2 m s^−1^, while wind speeds rising above 2 m s^−1^ appear to simply override any biological CO_2_ production and maintain the peat pore CO_2_ store at a low and constant level (Figure 4B). A similar threshold, at 2 m s^−1^ wind speed, was identified in experimental tunnels by Redeker and others (2015).

### Implications for Atmospheric Gas Exchange Measurements

The EC methodology can provide near-continuous measurements of atmosphere–biosphere CO_2_ exchange. When turbulent conditions prevail, the influence of wind on peat CO_2_ emission is continuously embedded in the EC-derived CO_2_ exchange measurements. However, during calm conditions, the air and peat below the EC system can accumulate and store considerable amounts of CO_2_, to be released at subsequent time steps, when turbulent conditions return. EC measurements of NEE are computed as the sum of the turbulent CO_2_ flux and this storage term (Baldocchi 2003). We estimate that accounting for the additional mass of CO_2_ that builds up in the peat during calm conditions nearly triples this storage term (Figure 6A). However, changes in CO_2_ storage flux tend to balance over the diel cycle (Figure 6B), and thus, their omission should not result in a significant bias in NEE over daily and longer timescales. Peat CO_2_ storage calculations should account for the full footprint of the EC measurements at Mer Bleue, given that the effect of wind on peat pore CO_2_ is similar in both hummock and hollow microforms of this peatland (average elevation difference 25 cm; Supplementary Figure S4) and the water table at the site lies well below the surface of both microforms throughout the summer (Supplementary Figure S1).

Many C cycle study applications require NEE partitioning to ER and GPP components. The ER component is typically modelled by quantifying the temperature dependence of night-time NEE (Reichstein and others 2005; Wutzler and others 2018) when there is no GPP, but also when lower wind speeds prevail (Figure 5C). The delays between peat CO_2_ production and emission caused by changes in wind speed throughout the day suggest that a uniform temperature extrapolation of night-time ER may not fully capture the diel dynamics in peat CO_2_ emissions, particularly from belowground sources, for example ER fluxes may be much higher than expected on windier nights as the peat CO_2_ store is vented to the atmosphere compared to calmer nights at the same temperature. The effect of changing wind speed on the peat CO_2_ store is most dynamic within the range of 0–2 m s^−1^ wind speeds, which dominates night-time measurements, indicating this effect could be most important over this specific period of the day. Although night-time EC measurements are typically filtered using friction velocity thresholds to help ensure only turbulent conditions are sampled, venting of the near-surface peat should be measured and accounted for using the rate of change in the storage term to ensure ER flux measurements represent biological processes.

Soil CO_2_ emissions are also commonly measured using soil chambers, where the effect of wind and pressure pumping has already received significant consideration (Xu and others 2006; Lai and others 2012). Our data indicate that wind effectively flushes the peat pore CO_2_ down to our lowest available measuring depth (− 40 cm in hummock and − 10 cm in hollows) (Figure 1, Supplementary Figure S3). Likely, wind can rapidly mobilize CO_2_ stored in the peat at least down to the water table. The collars of soil chambers in peatlands should extend down to the water table and factor in the change in effective chamber volume due to CO_2_ ventilation. Also, as noted by Lai and others (2012), chamber protocols may need to be modified at night. At Mer Bleue, chambers are closed for longer periods at night so that the initial emission rates are disregarded for steady-state conditions about 13 min or more after the chamber volume is sealed (Lai and others 2012). However, soil chamber measurements performed at different moments of the day may not fully capture the diel dynamics in peat CO_2_ transport with changing wind conditions. A possible solution to this issue might be to install adjustable fans inside the chambers to reflect ambient conditions.

### Implications for Ecosystem Processes and Modelling

Currently, peatland ecosystem models assume that soil respiration is instantly released to the atmosphere, thus omitting time–dynamic gas transport processes (for example, Parton and others 2010; Petrescu and others 2015; He and others 2021; Shao and others 2022). The modelled soil respiration is typically evaluated against the temperature-driven dynamics in ER from EC systems, which could lead to poor accuracy in modelled peat CO_2_ emissions over short timescales (sub-daily to daily, Figure 4). Wind predominantly affects the timing of peat CO_2_ emissions rather than gas production itself. Therefore, randomization of wind patterns over longer timescales (Zeng and others 2019) would likely mitigate this bias over multiple days to years. However, our data also indicate that together, wind speeds, temperature and soil moisture, influence changes in peat CO_2_ store across multiple days (Figures 2 and 4), for example hotter and dryer days enhance both peat venting and local CO_2_ production, while wetter and colder conditions dampen both processes (Figure 2). This triad of interconnected controlling factors (wind, temperature and moisture) may influence the accuracy of process-based models over multiple timescales.

Given that CO_2_ transport is many orders of magnitude faster in air compared with water, changes in soil moisture could play a dominant role in peat CO_2_ dynamics from daily to seasonal timescales. Intensification of the hydrological cycle, with increasing storm events and droughts, could amplify the future dynamics in peat CO_2_ emissions. Furthermore, changes in vegetation cover could also alter the natural surface roughness of an ecosystem and shift the influence of wind on peat CO_2_ dynamics. A more intermittent snow cover with lower thickness is anticipated in our studied region (Rafat and others 2021), which could leave the peat exposed to wind for longer periods of the year. At last, the potential changes in physical gas transport processes in response to water table restoration strategies (Evans and others 2021), aiming to reduce peat CO_2_ emissions (Page and Baird 2016; Ma and others 2022), may not have received sufficient attention. Overall, we suggest that the influence of wind on peat CO_2_ dynamics, within the context of changing peatland hydrology and plant community, be assessed in further detail to be incorporated across multiple aspects of peatland studies, including process-based models.

The role of peatlands as sources of atmospheric methane (CH_4_) has gathered significant interest in the context of short-term climate mitigation (Petrescu and others 2015). Non-diffusive methane fluxes are known to occur in peat porewater due to the low solubility of CH_4_ (for example, ebullition, plant-mediated transport and thermal convection) (Bellisario and others 1999; Tokida and others 2007; Poindexter and others 2016), but dynamics in the transport of free CH_4_ in peat are less known. The effect of wind on CH_4_ in air-filled peat pores could be manifested in two ways. Wind could enable CH_4_ to bypass methane oxidizer and allow a larger proportion of CH_4_ to reach the atmosphere (Clymo and Pearce 1997; Zheng and others 2018). Alternatively, peat methanogenesis could be suppressed by oxygen supplied through peat venting, decreasing peat CH_4_ emissions. A more detailed investigation of the interplay between CH_4_ production, oxidation and wind transport is recommended.

## Conclusions

Biological processes are often perceived as the dominant control over peat CO_2_ emissions because molecular diffusion is considered the main physical process through which CO_2_ is transported from the peat to the atmosphere. Our results demonstrate that dynamics in physical gas exchange dominate the short-term variability in peat CO_2_ store. Peat CO_2_ emission rates overwhelmingly exceed what could be attributed to molecular diffusion and vary considerably at the sub-daily and daily timescale, based on changes in wind speed. Consequently, peat CO_2_ production and emissions are not at a steady state but rather shifted in time because of dynamic non-diffusive transport processes. Venting of CO_2_ out of the peat influences the timing of peat CO_2_ emission and storage across several timescales. At the sub-daily timescale, peat venting suppresses the peat CO_2_ store during the daytime and enhances it at night. These effects can be reproduced across longer timescales, with changing wind regimes across different days. The influence of wind on peat CO_2_ dynamics blurs the physical boundary between the atmosphere and biosphere that is represented across many aspects of peatland studies. While peat venting could influence the accuracy of gap filling and modelling of EC-derived flux measurements together with the assessment of peat CO_2_ dynamics with other environmental factors at short timescales (sub-daily), its effect likely becomes negligible when integrated over sufficiently long timescales (multiple days to years). Nonetheless, our results suggest that our conceptualization of the peatland–carbon–climate nexus could overlook an important mechanism and thus call for a better comprehension of the physical transport processes that govern C cycling in peatlands.

### Supplementary Information

Below is the link to the electronic supplementary material.

Supplementary file1 (CSV 1976 kb)

Supplementary file2 (DOCX 2503 kb)

## Data Availability

Data is available in supplementary files.

## References

[CR1] Bahlmann LM, Smits KM, Heck K, Coltman E, Helmig R, Neuweiler I. 2020. Gas component transport across the soil-atmosphere interface for gases of different density: experiments and modeling. Water Resour Res 56.

[CR2] Baldocchi DD. 2003. Assessing the eddy covariance technique for evaluating carbon dioxide exchange rates of ecosystems: past, present and future. Glob Change Biol 9:479–492.

[CR3] Baldocchi D, Monson R. 2014. Wind and turbulence. Terrestrial Biosphere-Atmosphere Fluxes. Cambridge: Cambridge University Press. pp 296–326.

[CR4] Barba J, Cueva A, Bahn M, Barron-Gafford GA, Bond-Lamberty B, Hanson PJ, Jaimes A, Kulmala L, Pumpanen J, Scott RL, Wohlfahrt G, Vargas R. 2018. Comparing ecosystem and soil respiration: review and key challenges of tower-based and soil measurements. Agric for Meteorol 249:434–443.

[CR5] Bellisario LM, Bubier JL, Moore TR, Chanton JP. 1999. Controls on CH_4_ emissions from a northern peatland. Glob Biogeochem Cycl 13:81–91.

[CR6] Blodau C, Roulet NT, Heitmann T, Stewart H, Beer J, Lafleur P, Moore TR. 2007. Belowground carbon turnover in a temperate ombrotrophic bog. Glob Biogeochem Cycl 21:1–12.

[CR7] Bubier JL, Moore TR, Crosby G. 2006. Fine-scale vegetation distribution in a cool temperate peatland. Can J Botany 84:910–923.

[CR8] Campeau A, Vachon D, Bishop K, Nilsson MB, Wallin MB. 2021. Autumn destabilization of deep porewater CO_2_ store in a northern peatland driven by turbulent diffusion. Nat Commun 12:6857. 34824219 10.1038/s41467-021-27059-0 PMC8616934

[CR9] Clymo RS, Pearce DME. 1997. Methane and carbon dioxide production in, transport through, and efflux from a peatland. Philos Transact Royal Soc London Ser Phys Eng Sci 351:249–259.

[CR10] Davidson EA, Janssens IA. 2006. Temperature sensitivity of soil carbon decomposition and feedbacks to climate change. Nature 440:165–173. 16525463 10.1038/nature04514

[CR11] Dimitrov DD, Grant RF, Lafleur PM, Humphreys ER. 2010. Modeling the subsurface hydrology of Mer Bleue Bog. Soil Sci Soc Am J 74:680–694.

[CR12] Evans CD, Peacock M, Baird AJ, Artz RRE, Burden A, Callaghan N, Chapman PJ, Cooper HM, Coyle M, Craig E, Cumming A, Dixon S, Gauci V, Grayson RP, Helfter C, Heppell CM, Holden J, Jones DL, Kaduk J, Levy P, Matthews R, McNamara NP, Misselbrook T, Oakley S, Page SE, Rayment M, Ridley LM, Stanley KM, Williamson JL, Worrall F, Morrison R. 2021. Overriding water table control on managed peatland greenhouse gas emissions. Nature 593:548–552. 33882562 10.1038/s41586-021-03523-1

[CR13] He H, Jansson P-E, Gärdenäs AI. 2021. CoupModel (v6.0): an ecosystem model for coupled phosphorus, nitrogen, and carbon dynamics—evaluated against empirical data from a climatic and fertility gradient in Sweden. Geosci Model Dev 14:735–761.

[CR14] He H, Moore T, Humphreys ER, Lafleur PM, Roulet NT. 2023. Water level variation at a beaver pond significantly impacts net CO_2_ uptake of a continental bog. Hydrol Earth Syst Sci 27:213–227.

[CR15] Hirsch AI, Trumbore SE, Goulden ML. 2004. The surface CO_2_ gradient and pore-space storage flux in a high-porosity litter layer. Tellus B Chem Phys Meteorol 56:312–321.

[CR16] Koschorreck M, Knorr KH, Teichert L. 2022. Temporal patterns and drivers of CO_2_ emission from dry sediments in a groyne field of a large river. Biogeosciences 19:5221–5236.

[CR17] Lafleur PM, Roulet NT, Bubier JL, Frolking S, Moore TR. 2003. Interannual variability in the peatland-atmosphere carbon dioxide exchange at an ombrotrophic bog. Global Biogeochem Cycl 17.

[CR18] Lai DYF, Roulet NT, Humphreys ER, Moore TR, Dalva M. 2012. The effect of atmospheric turbulence and chamber deployment period on autochamber CO_2_ and CH_4_ flux measurements in an ombrotrophic peatland. Biogeosciences 9:3305–3322.

[CR19] Lerman. 1979. Geochemical Processes Water and Sediment Environments. Cambridge: Cambridge University Press.

[CR20] Lloyd J, Taylor JA. 1994. On the temperature dependence of soil respiration. Funct Ecol 315–323.

[CR21] Loisel J, Gallego-Sala AV, Amesbury MJ, Magnan G, Anshari G, Beilman DW, Benavides JC, Blewett J, Camill P, Charman DJ, Chawchai S, Hedgpeth A, Kleinen T, Korhola A, Large D, Mansilla CA, Müller J, van Bellen S, West JB, Yu Z, Bubier JL, Garneau M, Moore T, Sannel ABK, Page S, Väliranta M, Bechtold M, Brovkin V, Cole LES, Chanton JP, Christensen TR, Davies MA, De Vleeschouwer F, Finkelstein SA, Frolking S, Gałka M, Gandois L, Girkin N, Harris LI, Heinemeyer A, Hoyt AM, Jones MC, Joos F, Juutinen S, Kaiser K, Lacourse T, Lamentowicz M, Larmola T, Leifeld J, Lohila A, Milner AM, Minkkinen K, Moss P, Naafs BDA, Nichols J, O’Donnell J, Payne R, Philben M, Piilo S, Quillet A, Ratnayake AS, Roland TP, Sjögersten S, Sonnentag O, Swindles GT, Swinnen W, Talbot J, Treat C, Valach AC, Wu J. 2020. Expert assessment of future vulnerability of the global peatland carbon sink. Nat Clim Change 11:70–77.

[CR22] Ma L, Zhu G, Chen B, Zhang K, Niu S, Wang J, Ciais P, Zuo H. 2022. A globally robust relationship between water table decline, subsidence rate, and carbon release from peatlands. Commun Earth Environ 3.

[CR23] Massman WJ. 2006. Advective transport of CO_2_ in permeable media induced by atmospheric pressure fluctuations: 1. An analytical model. J Geophys Res 111.

[CR24] Moya MR, Lopez-Ballesteros A, Sanchez-Canete EP, Serrano-Ortiz P, Oyonarte C, Domingo F, Kowalski AS. 2022. Ecosystem CO_2_ release driven by wind occurs in drylands at global scale. Glob Chang Biol 28:5320–5333. 35727701 10.1111/gcb.16277 PMC9545467

[CR25] Orchard VA, Cook FJ. 1983. Relationship between soil respiration and soil moisture. Soil Biol Biochem 15:447–453.

[CR26] Page SE, Baird AJ. 2016. Peatlands and global change: response and resilience. Ann Rev Environ Resourc 41:35–57.

[CR27] Parton WJ, Hanson PJ, Swanston C, Torn M, Trumbore SE, Riley W, Kelly R. 2010. ForCent model development and testing using the enriched background isotope study experiment. J Geophys Res 115.

[CR28] Petrescu AM, Lohila A, Tuovinen JP, Baldocchi DD, Desai AR, Roulet NT, Vesala T, Dolman AJ, Oechel WC, Marcolla B, Friborg T, Rinne J, Matthes JH, Merbold L, Meijide A, Kiely G, Sottocornola M, Sachs T, Zona D, Varlagin A, Lai DY, Veenendaal E, Parmentier FJ, Skiba U, Lund M, Hensen A, van Huissteden J, Flanagan LB, Shurpali NJ, Grunwald T, Humphreys ER, Jackowicz-Korczynski M, Aurela MA, Laurila T, Gruning C, Corradi CA, Schrier-Uijl AP, Christensen TR, Tamstorf MP, Mastepanov M, Martikainen PJ, Verma SB, Bernhofer C, Cescatti A. 2015. The uncertain climate footprint of wetlands under human pressure. Proc Natl Acad Sci USA 112:4594–4599. 25831506 10.1073/pnas.1416267112 PMC4403212

[CR29] Phillips CL, Nickerson N, Risk D, Bond BJ. 2011. Interpreting diel hysteresis between soil respiration and temperature. Glob Change Biol 17:515–527.

[CR30] Poindexter CM, Baldocchi DD, Matthes JH, Knox SH, Variano EA. 2016. The contribution of an overlooked transport process to a wetland’s methane emissions. Geophys Res Lett 43:6276–6284.

[CR31] Poulsen TG, Furman A, Liberzon D. 2017. Effects of wind speed and wind gustiness on subsurface gas transport. Vadose Zone J 16.

[CR32] Pumpanen J, Ilvesniemi H, Hari P. 2003. A process-based model for predicting soil carbon dioxide efflux and concentration. Soil Sci Soc Am J 67:402–413.

[CR33] Rafat A, Rezanezhad F, Quinton WL, Humphreys ER, Webster K, Van Cappellen P. 2021. Non-growing season carbon emissions in a northern peatland are projected to increase under global warming. Commun Earth Environ 2.

[CR34] Rankin TE, Roulet NT, Moore TR. 2022. Controls on autotrophic and heterotrophic respiration in an ombrotrophic bog. Biogeosciences 19:3285–3303.

[CR35] Rappoldt C, Pieters G-JJM, Adema EB, Baaijens GJ, Grootjans AP, van Duijn CJ. 2003. Buoyancy-driven flow in a peat moss layer as a mechanism for solute transport. Proc Natl Acad Sci 100:14937–14942. 14657381 10.1073/pnas.1936122100 PMC299856

[CR36] Redeker KR, Baird AJ, Teh YA. 2015. Quantifying wind and pressure effects on trace gas fluxes across the soil–atmosphere interface. Biogeosciences 12:7423–7434.

[CR37] Reichstein M, Falge E, Baldocchi D, Papale D, Aubinet M, Berbigier P, Bernhofer C, Buchmann N, Gilmanov T, Granier A, Grunwald T, Havrankova K, Ilvesniemi H, Janous D, Knohl A, Laurila T, Lohila A, Loustau D, Matteucci G, Meyers T, Miglietta F, Ourcival J-M, Pumpanen J, Rambal S, Rotenberg E, Sanz M, Tenhunen J, Seufert G, Vaccari F, Vesala T, Yakir D, Valentini R. 2005. On the separation of net ecosystem exchange into assimilation and ecosystem respiration: review and improved algorithm. Glob Change Biol 11:1424–1439.

[CR38] Rey A. 2015. Mind the gap: non-biological processes contributing to soil CO_2_ efflux. Glob Change Biol 21:1752–1761. 10.1111/gcb.12821 25471988

[CR39] Roulet NT, Lafleur PM, Richard PJH, Moore TR, Humphreys ER, Bubier J. 2007. Contemporary carbon balance and late holocene carbon accumulation in a northern peatland. Glob Change Biol 13:397–411.

[CR40] Shao S, Wu J, He H, Roulet N. 2022. Integrating McGill wetland model (MWM) with peat cohort tracking and microbial controls. Sci Total Environ 806:151223. 34717989 10.1016/j.scitotenv.2021.151223

[CR41] Smith B, Wårlind D, Arneth A, Hickler T, Leadley P, Siltberg J, Zaehle S. 2014. Implications of incorporating N cycling and N limitations on primary production in an individual-based dynamic vegetation model. Biogeosciences 11:2027–2054.

[CR42] Spohn M, Holzheu S. 2021. Temperature controls diel oscillation of the CO_2_ concentration in a desert soil. Biogeochemistry 156:279–292.

[CR43] Stoica P. aMR. 1997. Introduction to spectral analysis.

[CR44] Teklemariam TA, Lafleur PM, Moore TR, Roulet NT, Humphreys ER. 2010. The direct and indirect effects of inter-annual meteorological variability on ecosystem carbon dioxide exchange at a temperate ombrotrophic bog. Agric for Meteorol 150:1402–1411.

[CR45] Tokida T, Miyazaki T, Mizoguchi M, Nagata O, Takakai F, Kagemoto A, Hatano R. 2007. Falling atmospheric pressure as a trigger for methane ebullition from peatland. Glob Biogeochem Cycl 21: n/a-n/a.

[CR46] Treat CC, Kleinen T, Broothaerts N, Dalton AS, Dommain R, Douglas TA, Drexler JZ, Finkelstein SA, Grosse G, Hope G, Hutchings J, Jones MC, Kuhry P, Lacourse T, Lahteenoja O, Loisel J, Notebaert B, Payne RJ, Peteet DM, Sannel ABK, Stelling JM, Strauss J, Swindles GT, Talbot J, Tarnocai C, Verstraeten G, Williams CJ, Xia Z, Yu Z, Valiranta M, Hattestrand M, Alexanderson H, Brovkin V. 2019. Widespread global peatland establishment and persistence over the last 130,000 y. Proc Natl Acad Sci USA 116:4822–4827. 30804186 10.1073/pnas.1813305116 PMC6421451

[CR47] Wickham H, Averick M, Bryan J, Chang W, McGowan L, François R, Grolemund G, Hayes A, Henry L, Hester J, Kuhn M, Pedersen T, Miller E, Bache S, Müller K, Ooms J, Robinson D, Seidel D, Spinu V, Takahashi K, Vaughan D, Wilke C, Woo K, Yutani H. 2019. Welcome to the Tidyverse. J Open Source Softw 4.

[CR48] Wörman A, Lindström G, Riml J. 2017. The power of runoff. J Hydrol 548:784–793.

[CR49] Wu J, Roulet NT, Nilsson M, Lafleur P, Humphreys E. 2012. Simulating the carbon cycling of northern peatlands using a land surface scheme coupled to a wetland carbon model (CLASS3W-MWM). Atmos Ocean 50:487–506.

[CR50] Wutzler T, Lucas-Moffat A, Migliavacca M, Knauer J, Sickel K, Šigut L, Menzer O, Reichstein M. 2018. Basic and extensible post-processing of eddy covariance flux data with REddyProc. Biogeosciences 15:5015–5030.

[CR51] Xu L, Furtaw MD, Madsen RA, Garcia RL, Anderson DJ, McDermitt DK. 2006. On maintaining pressure equilibrium between a soil CO_2_ flux chamber and the ambient air. J Geophys Res 111.

[CR52] Yvon-Durocher G, Caffrey JM, Cescatti A, Dossena M, del Giorgio P, Gasol JM, Montoya JM, Pumpanen J, Staehr PA, Trimmer M, Woodward G, Allen AP. 2012. Reconciling the temperature dependence of respiration across timescales and ecosystem types. Nature 487:472–476. 22722862 10.1038/nature11205

[CR53] Zeng Z, Ziegler AD, Searchinger T, Yang L, Chen A, Ju K, Piao S, Li LZX, Ciais P, Chen D, Liu J, Azorin-Molina C, Chappell A, Medvigy D, Wood EF. 2019. A reversal in global terrestrial stilling and its implications for wind energy production. Nature Clim Change 9:979–985.

[CR54] Zhang Q, Katul GG, Oren R, Daly E, Manzoni S, Yang D. 2015. The hysteresis response of soil CO_2_ concentration and soil respiration to soil temperature. J Geophys Res Biogeosci 120:1605–1618.

[CR55] Zheng J, RoyChowdhury T, Yang Z, Gu B, Wullschleger SD, Graham DE. 2018. Impacts of temperature and soil characteristics on methane production and oxidation in Arctic tundra. Biogeosciences 15:6621–6635.

