# Supplementary material for: Wind as a Driver of Peat CO2 Dynamics in a Northern Bog
Source: Ecosystems. 2024 May 23;27(5):621–35. doi: 10.1007/s10021-024-00904-1 (PMC11289004; doi:10.1007/s10021-024-00904-1)
Supplement: Supplementary file 2 — Supplementary file2 (DOCX 2503 kb) [file 10021_2024_904_MOESM2_ESM.docx]

**Supplementary Files**

**Figure S1**: Timeseries of water table depth below ground surface at the site between June to December 2009.


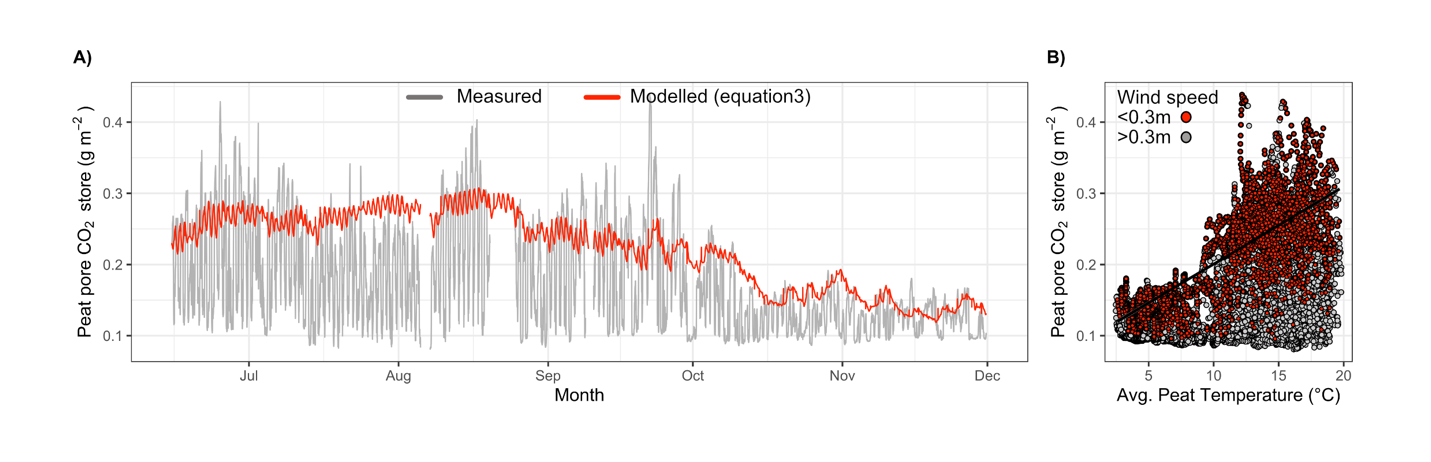


**Figure S2:** A) Time series of peat pore CO_2_ store between June and December 2009 (grey), with the red line representing the time series of peat pore CO_2_ store estimated by equation 3. In B) peat pore CO_2_ store as a function of the average peat temperature (5 to 40cm) when wind speed measured 2.6m above ground surface is >0.3m (grey) or <0.3m (red). In B) the thick grey line represents the regression line for equation 3.


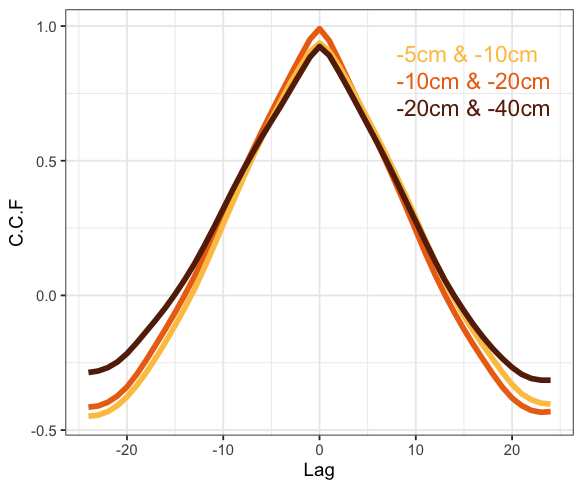


Figure S3: Cross correlation plot of peat pore pCO_2_ at different depths indicating high positive correlation at 0 time lag along the vertical depth profile.

**Figure S4**: Cross correlation plots of peat temperature and CO_2_ concentration (ppm) from June to December 2009 at (a) 5 cm, (b) 10 cm, (c) 20 cm and (d) 40 cm below ground surface, with time lag raging from –24 to +24 hrs. Highest correlation occurring at time lag <0 indicate that peat temperature leads CO_2_ concentration in time, while those occurring at time lag>0 indicate that peat temperature lags CO_2_ in time.

Figure S5: Scatterplot of peat pore CO_2_ concentration and temperature measured a different depth from the ground surface (5cm yellow, 10cm orange, 20cm dark orange, 40cm brown). Each panel represent a different day in July 2009 (n=30 days). Each point represents a half-hour measurement linked together by a colored line (n=48 observations per panel).


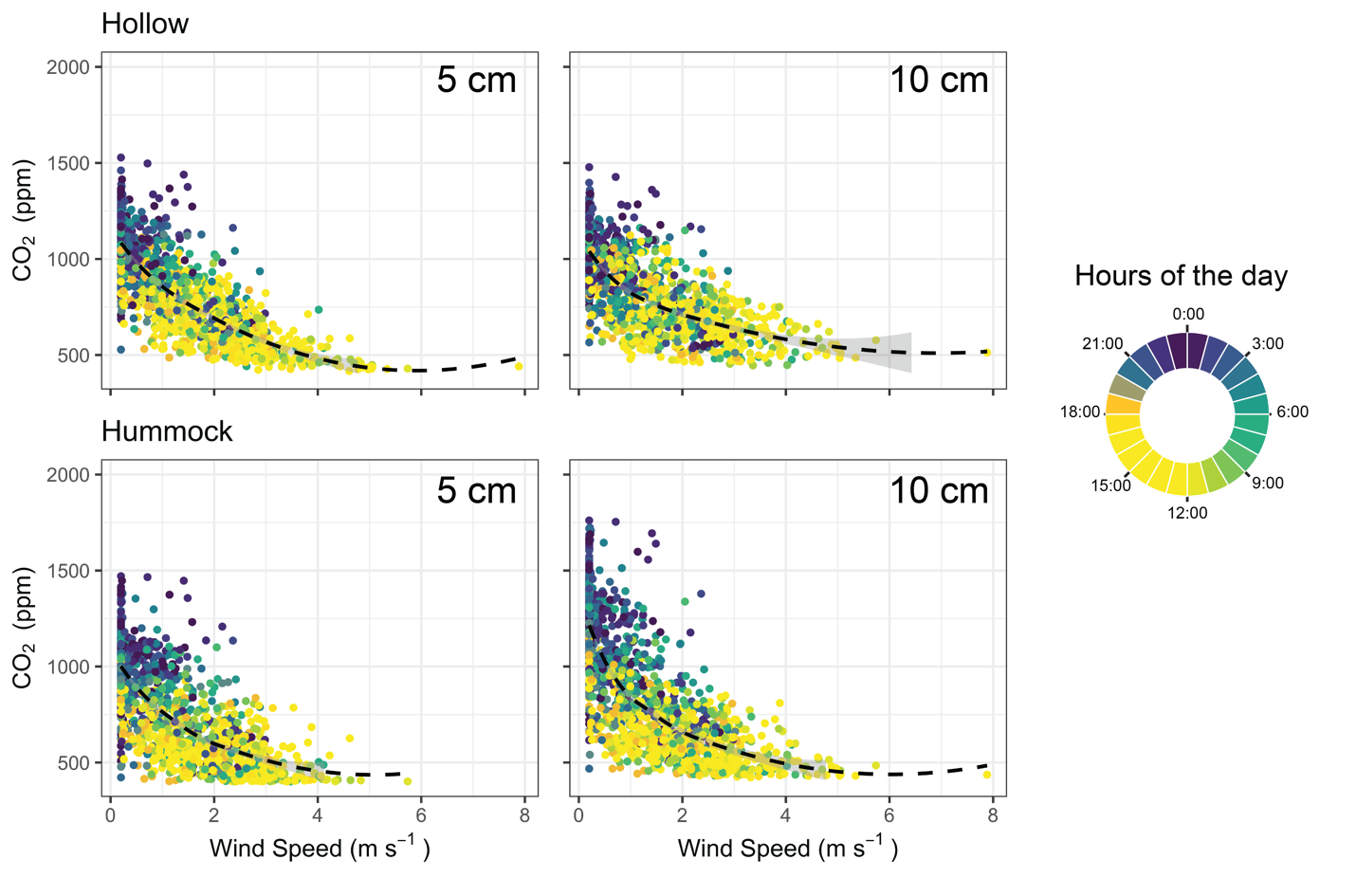


**Figure S6**: Loess regression model between wind speed and peat pore CO_2_ concentration (ppm) at 5cm (left) and 10cm (right) below ground surface in a hollow (top) and a hummock (bottom) microform at half-hour intervals in July 2009.
